# Developing Fluorescent Hyaluronan Analogs for Hyaluronan Studies

**DOI:** 10.3390/molecules17021520

**Published:** 2012-02-07

**Authors:** Wei Wang, Arlin G. Cameron, Shi Ke

**Affiliations:** Department of Radiology, Baylor College of Medicine, Houston, TX 77030, USA; Email: ww1@bcm.edu (W.W.); arlin.g.cameron@uth.tmc.edu (A.G.C.)

**Keywords:** hyaluronan, hyaluronidase, imaging agent, biosensitive imaging agent

## Abstract

Two kinds of fluorescent hyaluronan (HA) analogs, one serving as normal imaging agent and the other used as a biosensitive contrast agent, were developed for the investigation of HA uptake and degradation. Our approach of developing HA imaging agents depends on labeling HA with varying molar percentages of a near-infrared (NIR) dye. At low labeling ratios, the hyaluronan uptake can be directly imaged while at high labeling ratios, the fluorescent signal is quenched and signal generation occurs only after degradation. It is found that the conjugate containing 1%-2% NIR dye can be used as a normal optical imaging agent, while bioactivable imaging agents are formed at 6% to 17% dye loading. It was determined that the conjugation of dye to HA with different loading percentages does not impact HA biodegradation by hyaluronidase (Hyal). The feasibility of using these two NIR fluorescent hyaluronan analogs for HA investigation was evaluated *in vivo* with optical imaging. The data demonstrates that the 1% dye loaded fluorescent HA can be used to monitor the behavior of HA and its fragments, whereas bioactivatable HA imaging agent (17% dye in HA) is more suitable for detecting HA fragments.

## 1. Introduction

Hyaluronan (HA), also known as hyaluronic acid, is a high molecular weight, negatively charged linear polyglycosaminoglycan, consisting of repeating units of the disaccharide D-glucuronic acid-ß(1→3)-*N*-acetyl-D-glucosamine-ß(1→4) [1,2,3]. HA is formed on the inner face of the plasma membrane of fibroblasts, directly extruded into the extracellular matrix, and distributed in nearly all tissues. The highest HA concentrations are found in soft connective tissues (human umbilical cord) and the lowest in blood serum [4]. The polysaccharide acts as a lubricant and is associated with processes in which cell migration is involved [5,6]. HA catabolism occurs through three different degradation pathways. The hyaluronidase (Hyal) enzyme family appears to be prominent in HA catabolism [2,7]. It is known that hyaluronan is related with different kinds of diseases. For example, elevated HA, HA synthase, and Hyal activity are all associated with cancer cells and cancer-associated tissue stroma, consistent with their roles for mediating cell migration and metastasis at various stages of disease progression [8,9]. HA oligomers formed by Hyal degradation are proangiogenic [10,11] and have inflammatory and immuno-stimulatory properties [12]. The HA clearance ability of liver can indicate the functional status of the general health of the liver, which is a useful parameter for predicting the success of human liver transplantation [13,14]. Furthermore, the physical properties and biocompatibility of hyaluronan also make this polymer considerably important in the development of engineered tissue, biomaterials and in clinical applications [15]. Therefore, a complete understanding of HA uptake and its degradation can help to better understand their significant roles under normal and pathological condition. Here we report the development of an optical imaging system for the study of hyaluronan behaviors *in vivo* with two kinds of florescent hyaluronan analogs, one being normal optical imaging agent and the other one bioactivatable imaging agent, with the latter being a quenched HA complex capable to beacon fluorescence due to surrounding bioenvironmental changes. HA labeling with radio-iostopes has previously been used to image the distribution of HA uptake [16,17,18]. However, we take advantage of NIR imaging to provide a method for tracking HA behaviors with fluorescent labels without a physical half-life as radio-isotope and with high sensitivity for small animal imaging.

## 2. Results and Discussion

### 2.1. Chemistry

The goal of this study was to develop a particular system for evaluating HA functionality from the protein level to the whole animal level using an optical molecular imaging technique. To achieve this goal the strategy used was to design two different kinds of fluorescent HAs which are employed as HA substrate analogs and can be monitored by optical imaging. One of them is a normal imaging agent, which can be used to study both HA and its fragments. The other one functions as a bioactivable imaging probe which is optically silent (quenched) in its original state and emits fluorescence through enzyme degradation. In contrast to normal optical imaging agent, the latter is employed for imaging HA fragments only and providing information on the location of the degradation caused by the presence of enzymes. Therefore, we can visualize real time HA uptake and its degradation *in vivo* using optical imaging by means of these two contrast agents. This system provides detailed information about HA functions, circulation, distribution, degradation and clearance *in vivo*.

The design of these fluorescent HA imaging agents was based on the fact that varying molar ratio between dye and HA polymer can change fluorescence intensity [19,20,21,22]. Attachment of dye molecules covalently to HA results in fixed distance between dyes on a single HA polymer. When a certain dye molar ratio to HA is reached, fluorophores are close enough so that they quench each other by mutual energy transfer (overlapped excitation and emission spectrum). It makes the fluorescence intensity of the conjugate decrease. The effect can be strong enough that visual signal even becomes undetectable. These compounds are fluorescently quenched in their native states. The fluorescence signal will be generated by the compound degradation, whereby the long chains of polymer are split into shorter ones, the fixed distances between dyes are broken, leading to the fluorescence intensity recovery. Therefore, bioactivable imaging agents can be formed in such dye loading percentage ranges.

**Scheme 1:**
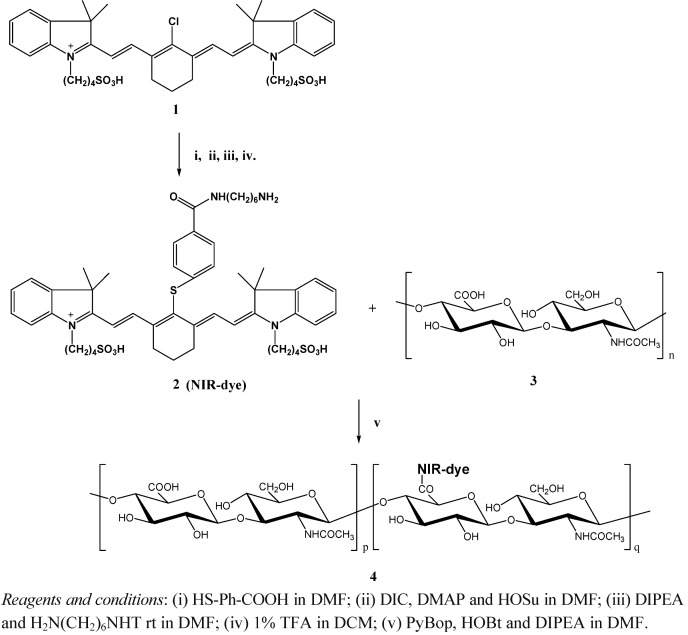
Synthesis of fluorescent hyaluronan.

**Figure 1:**
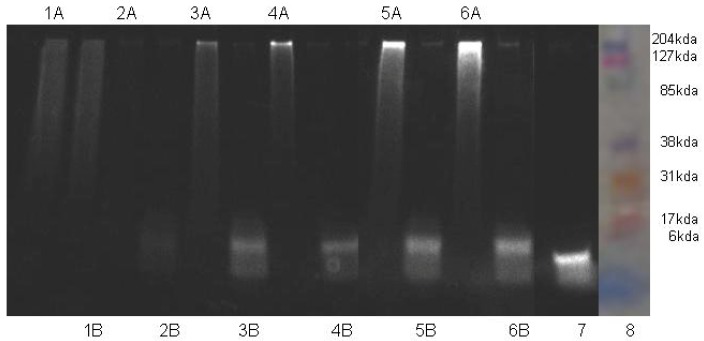
Characterization of HA-dye conjugates using SDS- PAGE assay. (**1**) 15% dextran-dye; (**2**) 90% HA-dye; (**3**) 17% HA-dye; (**4**) 9% HA-dye; (**5**) 4% HA-dye; (**6**) 1% HA-dye; (**7**) NIRdye; (**8**) molecular weight maker; (**A**) without hyaluronidase; (**B**) with hyaluronidase.

Since NIR light (wavelength range of 700-900 nm) possesses relatively low autofluorescence and is minimally absorbed by hemoglobin as well as by water and lipids, NIR fluorophore [NIR-dye **2** (Figure 1)] was selected as NIR fluorescence contrast for this study. To achieve both efficient fluorescence signal quenching and sufficient fluorescence signal recovering by enzymatic degradation, a series of HA-NIRdye conjugates with different dye loading percentages were designed. All fluorescent HA complexes were prepared through conjugation of NIRdye **2** to hyaluronan via an amide transformation as shown in Scheme 1. Non-specific, dextran-NIRDye conjugate as negative control was prepared in a similar synthetic way.

### 2.2. Characterization

The purities of HA-NIRdye conjugates and their degradation level by hyaluronidase were analyzed using SDS-PAGE method (Figure 1). Two groups (A and B) of HA-dye conjugates with different dye loadings, one (A) without and the other (B) with enzyme hyaluronidase incubation were prepared for this assay. Pure dye **2** was used as a positive control (Figure 1, lane 7). The other polysaccharide (dextran) conjugated with 15% loading NIRdye served as a negative control (Figure 1, lanes 1A and 1B). The results demonstrate that synthetic conjugates are relatively pure since no trace of free dye is detected and no low molecular weight HA-dyes exist (Figure 1, A lanes). It is found that there is no macromolecule left for all of HA-dye conjugates after 24 h incubation with hyaluronidase (Figure 1, lanes 2B through 6B for HA-NIRdye with 90, 17, 9, 4, and 1 molar percent loading of dye), while no biodegradation of dextran-NIRdye conjugate in the presence of hyaluronidase is observed over the 24-h period (Figure 1, lane 1B). The data indicate that the conjugation of dye to HA with different loading percentages, even at 90%, does not influence HA degradation by Hyal. Fluorescently labeled HAs retain their biological activity. The 90% HA-NIRdye shows little fluorescence in either the intact or degraded states, consistent with the substantial quenching at high molar loadings of dye (see below). It is also noteworthy that as the molar loading of NIR dye decreases, the fluorescent intensity of the electrophoresed bands increases, consistent with the loss of quenching.

**Figure 2:**
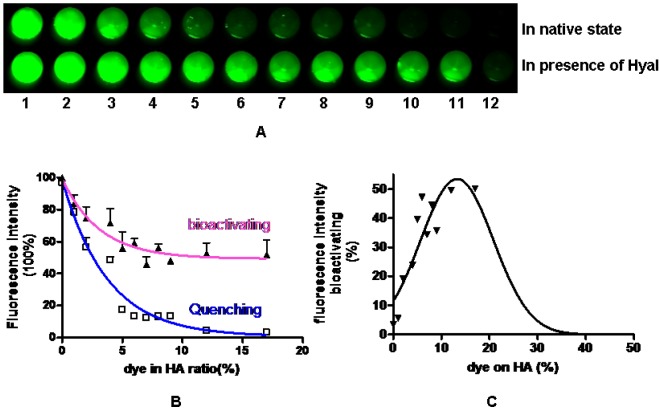
Fluorescence intensity of HA-dye conjugates in native state and in presence of enzyme hyaluronidase (**A**) fluorescence intensity of each conjugate without (top) and with Hyal (bottom) The compounds in wells are (**1**) dye; (**2**) HA-dye 1%; (**3**) HA-dye 2%; (**4**) HA-dye 4%; (**5**) HA-dye 5%; (**6**) HA-dye 6%; (**7**) HA-dye 7%; (**8**) HA-dye 8%; (**9**) HA-dye 9%; (**10**) HA-dye 12%; (**11**) HA-dye 17%; (**12**) HA-dye 90%; (**B**) The curves represent fluorescence intensity quenching and bioactivating effects as functions of dye loading percentage on HA; (**C**) The curve depicts the recovering fluorescence intensity of HA-dye conjugates.

In order to test the degradation capability of Hyal, we determined the quenching efficiency quantitatively through measurement of fluorescent intensity. Figure 2A pictorially represents the percentage of the fluorescence intensities of the HA-NIRdye conjugates in their native state and after 24 h degradation in the presence of Hyal. Figure 2B graphically illustrates the percentage of fluorescent intensities relative to an equivalent amount of free, unquenched NIRdye (**2**) as a function of molar dye loading. The results show that signal intensity of 1% HA-NIRdye is approximately 81% due to an equivalent amount of unconjugated NIRdye (**2**). Upon increasing the dye loading percentage to 6%, substantial dye quenching occurrs reducing the signal to 18% due to an equivalent amount of NIRdye (**2**). Almost complete quenching (5% intensity remaining) was reached when molar ratio of NIRdye to HA was over12%.

Figure 2B shows that the molar percentage of dye loading is a critical factor for beacon performance and that the brightest intact “contrast agent” has the lowest dye loading. At a lower dye loading (1%), the fluorescence intensity of the conjugate remains comparatively unchanged after degradation by Hyal, indicating the agent’s inability to report degradation, albeit having the greatest efficiency for reporting HA uptake owing to its greatest fluorescent yield. Upon increasing the molar dye loading from 1 to 6%, the fluorescence intensity change is significantly enhanced by degradation. 

Figure 2C is a plot of the percentage change of fluorescent intensity upon Hyal incubation for 24 h as a function of percentage of molar dye loading and shows that maximum, full quench is reached by dye loading between 12 and 17%. At the loading percentage of 90%, the fluorescence signal remained fully quenched even following degradation. The SDS-PAGE result readily demonstrates that HA-NIRdye conjugates were totally split into fragments, even at the highest loading percentage. On the other hand, it is known that the smallest HA fragments cleaved by hyaluronidase are tetrasaccharides [23]. Thus, neither polymer nor monomer, rather only oligomer with molecular weight below 6,000 kda existed after degradation by Hayl. We conclude that dye loading is substantially high so that on average more than one dye are attached to a single HA fragment, so that fluorescence signal will not be able to fully recover or even remain quenched.

**Figure 3:**
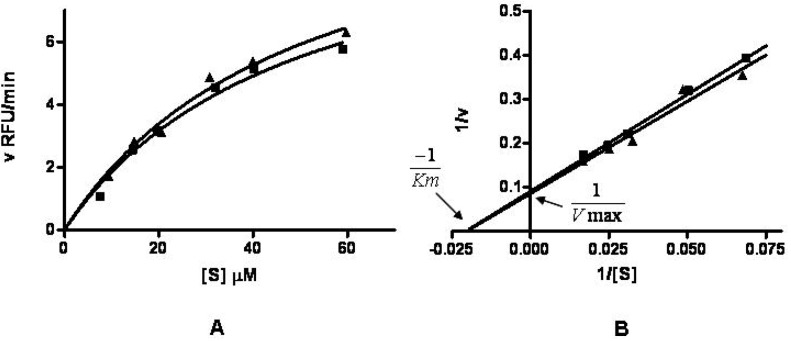
Degradation of HA-NIRdye conjugates in presence of Hyal. (**A**) Degradation of HA-NIRdyes (■ 17%and ▲ 12%) by Hyal can be depicted by Michaelis-Menten kinetics (RFU: relative fluorescence units); (**B**) Michaelis-Menten kinetics of degradation of HA-NIRdyes (■ 17%and ▲ 12%) can be displayed on Lineweaver-Burk plot.

Because the HA-NIRdyes conjugates at dye loading percentages of 12 and 17% exhibited excellent efficiency for both fluorescence quenching and bioactivation, we further examined their sensitivity to enzymatic Hyal activity. Figure 3A,B illustrate the initial reaction velocity, v (RFU/min), as a function of conjugate concentration for 12 and 17%. It is found that the degradation mechanisms of both compounds in presence of Hyal can be described by Michaelis-Menten kinetics either using nonlinear regression with Michaelis-Menten equation (Figure 3A) [24] or fitting the data to Lineweaver-Burk plot (Figure 3B) [25]. The Michaelis-Menten constants Km for 17% conjugate and 12% complex are 48.5 and 49.2 μM, respectively.

**Figure 4:**
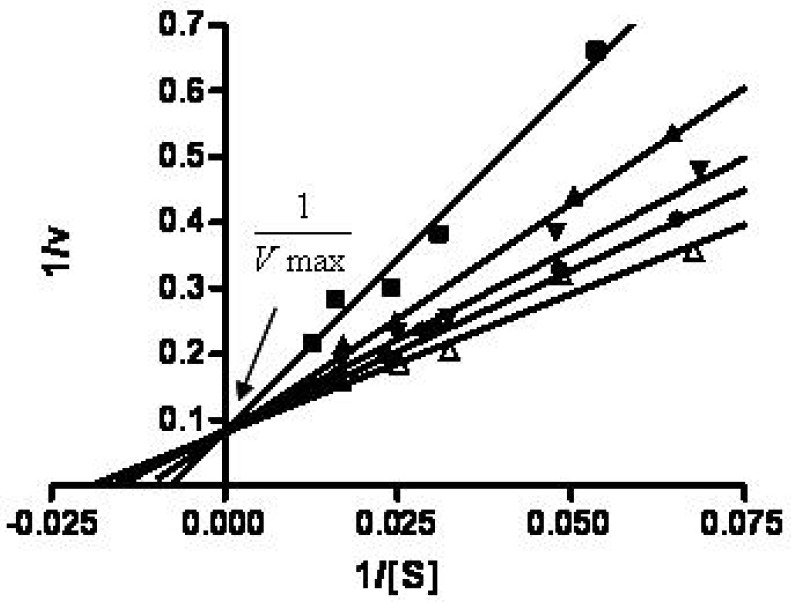
Lineweaver-Burk plots of 12% HA-NIRdye degradation catalyzed by Hyal with normal HA as inhibitor (inhibitor concentrations ∆ 0 μM, ● 16.5 μM, ▼33 μM, ▲ 66 μM and ■ 100 μM).

To further evaluate whether the HA-NIRdye conjugates retained similar biological activity to HA, competitive inhibition studies were performed. We employed HA as inhibitor and incubated it with 12% HA-NIRdye and hyaluronidase together. Figure 4 illustrates a set of typical Linearweaver-Burk plots of reciprocal initial reaction velocity as a function of reciprocal 12% HA-NIRdye concentration with HA at concentrations of 0, 16.5, 33, 66, and 100 μM. That Vmax is shown remaining unchanged at different concentrations of HA in Figure 4. This evidenced that HA-NIRDye binds to the same active site of the enzyme Hyal as HA. These results further reinforce the inference that the biological activity of HA-NIRdye conjugates was not weakened.

### 2.3. *In Vivo* Imaging of 1 and 17% HA-NIRDye Conjugates

**Figure 5:**
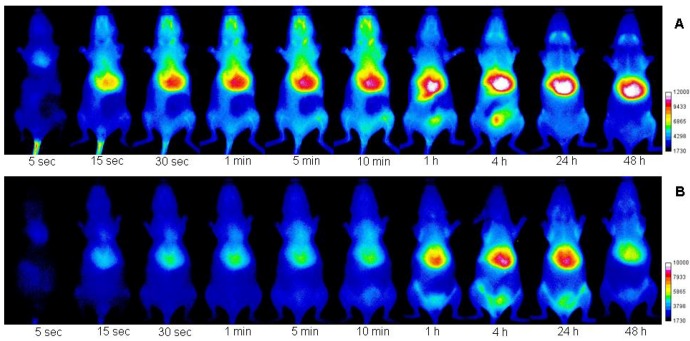
Optical images of SKH mice injected with fluorescent HAs (A: 1% and B: 17%) at different time points: 5, 15, 30 s, 1, 5, 10min, 1, 4, 24, 48 h. Figure shows the same mouse imaging results over 48 h time period.

**Figure 6:**
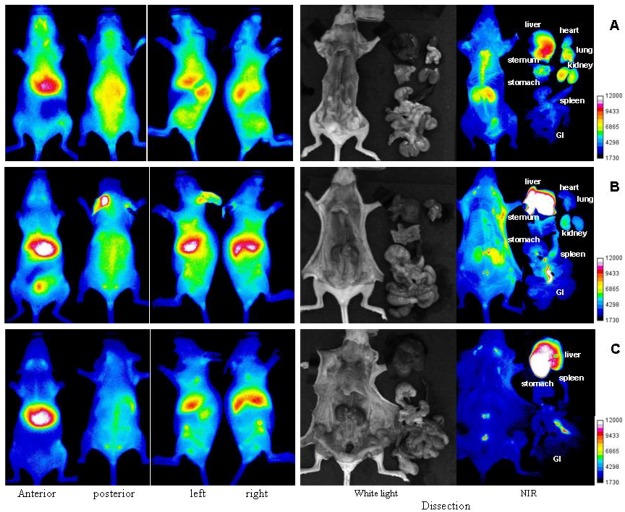
Representative optical imaging results of SKH mice injected with 1% HA-NIRdye at different time points: (**A**) 10min, (**B**) 4 h and (**C**) 48 h.

**Figure 7:**
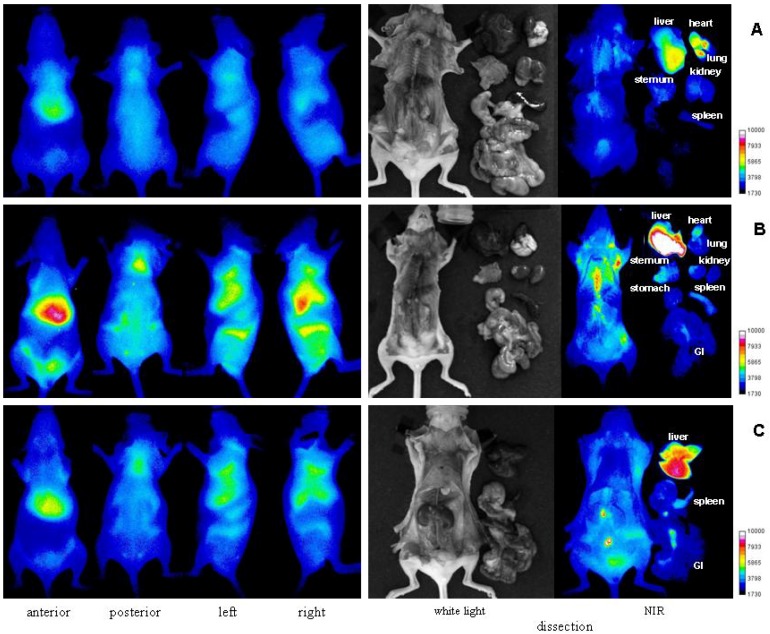
Representative optical imaging results of SKH mice injected with 17% HA-NIRdye at different time points: (**A**) 10 min, (**B**) 4 h and (**C**) 48 h.

To investigate the use of HA-NIRDye for imaging HA uptake and degradation, we chose to conduct *in vivo* imaging using conjugates with 1 and 17% molar percent loading of dye. Fluorescent HAs with same dye molar concentration were injected intravenously into SKH mice. Their dynamic imaging was carried out for 10 min. The whole body imaging continued at predetermined time intervals during the next 48 h. The *in vivo* imaging pictures at different time points after injection with these two agents are shown in Figure 5A (1%) and Figure 5B (17%), where both labeled HAs’ behavior *in vivo* can be followed and compared with each other over the imaging period. More detailed information for each compound at 10 min, 4 h and 48 h is illustrated in Figure 6 or Figure 7.

It is found in real time imaging that the fluorescence signal was registered in the tail vein immediately and in the whole body just a few minutes after intravenous injection of normal fluorescent HA (1% dye loading) from tail vein (Figure 5A and Figure 6A). Unlike the 1% fluorescent HA-NIRDye, there is almost no fluorescent signal detected for the bioactivable fluorescent HA (17% dye loading) at the tail vein injecting position due to the quenching effect (Figure 5B). However, the fluorescence intensity of this labeled HA started increasing, especially at liver and lung positions, rapidly after the injection due to bioactivation (Figure 5B and Figure 7A). The results indicate that the labeled HAs swiftly distributed to the whole body through the circulation after injection and a rapid degradation happened once the compound entered the circulation. The degradation of 17% HA-dye was clearly visualized in whole body at 1 h after injection. The fluorescence reached high intensity at 4 h and continues to 24 h after injection and slowly declined over the next 24 h. It is well known that structure change will certainly alter the parent compound's biological properties. The 17% HA-dye may have different binding affinity than 1% HA-dye. The peak signal intensity time delay can not be excluded from the possibilities of other receptor binding or receptor-mediated events in the *in vivo* studies.

Detailed information of uptake in different organs for each compound was obtained after dissections, which were carried out at 10 min, 4 h and 48 h time period after injection of these two optical conjugates respectively. The results are depicted in the Figure 6 for 1% HA-dye and Figure 7 for 17% HA-dye, where a complementary picture of organs uptake of HA was provided.

It is clearly visualized that a major portion of both labeled HA was taken up by the liver which is responsible for the elimination of most HA in the circulation. Besides in liver, it is found that fluorescent HAs existed in the kidney, lung, heart, skeletal, spleen, various lymph nodes and bone marrow. These findings confirm the reported observations by Fraser [26] using autoradiographic method.

Furthermore, it is found that the kidney uptake of HA is only visualized in normal fluorescent HA dissection by comparing Figure 6 and Figure 7, indicating that polymer HA may be the only compound which was extracted in kidney. It is known that the kidney is relatively rich in degrading enzymes [27]. The fact no HA fragments were detected may be because HA fragments were removed rapidly from kidney once degradation occurred. Except for kidney, all other organs’ uptake of HA is reported in both labeled HA dissections. It points out that at least HA fragments existed in all these organs. It is noticed that uptake amounts of HA and/or its fragments in different organs were different functions of time, e.g., notable amount of fluorescence were detected in kidney and lung within a short period (4 h), whereas HA accumulation in the spleen or intestinal lymph is a rather time consuming process.

The retention of fluorescence in the lymph nodes suggests that there is a high affinity for HA and its fragments in lymph nodes. In addition, optical imaging also demonstrates that the fluorescence intensities have been varied depending on the lymph species. The highest intensity was shown in intestinal lymph (Figure 6C and Figure 7C). The visualization capability of labeled HAs and their fragments in lymph provides an opportunity for detection and resection of lymph nodes.

In this work we proved that the bioactivatable NIR-HA is useful for in vivo imaging of gross changes in HA metabolism. It remains to be investigated whether it is sensitive to more subtle changes that involve HA interaction with HA receptors and binding proteins including CD44, LYVE-1, Stabilin 2/HARE, TSG-6, aggrecan, versican, Link protein, *etc*.

## 3. Experimental

### 3.1. Materials

*N,N*-diisopropylethylamine (DIPEA), IR-783, 4-mercaptobenzoic acid, 4-dimethylaminopyridine (DMAP), *N*-hydroxysuccinimide (HOSu), 1,3-diisopropylcarbodiimide (DIC), hyaluronic acid potassium salt from human umbilical cord and hyaluronidase type 1-s were purchased from Sigma-Aldrich (St. Louis, MO, USA). Dextran amino was purchased from Invitrogen (Carlsbad, CA, USA). Hydroxybenzotriazole (HOBt), mono-trityl-1,6-diaminohexane acetic acid salt and benzotriazol-1-yl-oxy-tris-pyrrolidinophosphonium hexaflurophosphate (PyBOP) were purchased from Novabiochem (San Diego, CA, USA). All solvents were purchased from VWR (San Dimas, CA, USA).

### 3.2. Analysis

Analytical high-performance liquid chromatography (HPLC) was performed on an Agilent 1100 HPLC system equipped with a Varian reverse phase C-18 analytical column (R0086200CG) at a flow rate of 1 mL/min. Samples were eluted with H_2_O/acetonitrile containing 0.1% TFA using linear gradient 10% to 80% in 30 min. Matrix-assisted laser desorption ionization mass spectrometry (MALDI) and electrospray ionization mass spectrometry (ESI) were performed by the Protein Chemistry Core Laboratory at Baylor College of Medicine. Fluorescence intensities were detected by an Odyssey Near-Infrared Imaging System from LiCor (Lincoln, NE, USA) and GENios Pro from Tecan (San Jose, CA, USA). Optical imaging was performed with an electron-multiplying charge-coupled device (EMCCD) camera (model PhotoMAX:512B, Princeton Instruments, Trenton, NJ, USA).

### 3.3. Synthesis of NIR-dye [2-((*E*)-2-((*E*)-2-(4-(6-aminohexylcarbamoyl)phenylthio)-3-((*E*)-2-(3,3-dimethyl-1-(4-sulfobutyl)indolin-2-ylidene)ethylidene)cyclohex-1-enyl)vinyl)-3,3-dimethyl-1-(4-sulfobutyl)-3*H*-indolium] (**2**)

IR-783 (**1**) was reacted with 4-mercaptobenzoic acid to produce IR-783-S-Ph-COOH (i) [28], which was converted to its active ester by treatment with HOSu (1.5 equiv.) in the presence of DIC (1.5 equiv.) and DMAP (0.2 equiv.) in DMF (ii). The further acylation was carried out with mono-trityl-1,6-diaminohexane acetic acid salt (1 equiv.) in DMF with 10% of DIPEA (iii). The amino-NIR-dye 2 was formed after removal of trityl- protecting groups using 1% trifluoacetic acid (TFA) in DCM (iv). The product from each step was purified by flash chromatography eluted with ethyl acetate and methanol. The structure of compound **2** was verified by mass spectroscopy (M^+^ 943.42 [calculated], 943.40 [found]) and HPLC (retention time 19.85min).

### 3.4. Synthesis of a Series of HA-NIRdye

The amount of NIR-dye **2** used for each HA-dye conjugate was calculated according to the desired molar ratio of dye to monomer of HA. NIR-dye **2** was coupled to the carboxylic acid groups of HA (**3**) (Figure 1) in the presence of PyBOP (1 equiv.), HOBt (2 equiv.) and DIPEA (4 equiv.) in DMF. The conjugate was purified by washing with MeOH. HA-NIRdye conjugates were not dissolved in MeOH. All the filtrates were collected together for evaluation of unreacted dye using HPLC. The conjugate products were dissolved in water and lyophilized. The molar dye loading amount for each batch was calculated by subtracting the amount of unreacted dye from total dye amount for reaction. The purity of each product was examined by SDS-PAGE Analysis. The HA-dye conjugates were identified by their molar percentage of dye to monomer content.

### 3.5. Synthesis of 15% Dextran-NIRdye

The amount of IR-783-*S*-Ph-COOH used for dextran-dye conjugate was calculated according to the desired molar ratio of dye to monomer of dextran. IR-783-S-Ph-COOH was coupled to the amino groups of dextran in the presence of PyBOP (1 equiv.), HOBt (2 equiv.) and DIPEA (4 equiv.) in DMF. The conjugate was purified by washing with MeOH. All the filtrates were collected together for evaluation of unreacted dye using HPLC. The product was dissolved in water and lyophilized. The molar dye loading amount for dextran was calculated by subtracting the amount of unreacted dye from total dye amount for reaction. The purity of product was examined by SDS-PAGE Analysis.

### 3.6. SDS-PAGE Analysis

The stock solutions (80 μM) for HA-NIRdye conjugates, 15% dextran-NIRdye and NIRdye **2** were prepared in buffer A (300 mM sodium phosphate pH 5.35) respectively. Two assays of each compound were carried out, one in the presence of Hyal and the other without. For the former, a compound (50 μL stock solution) was incubated with hyaluronidase (25 units) in 50 μL buffer B (20 mM sodium phosphate, 77 mM sodium chloride and 0.01% (w/v) bovine serum albumin, pH 7.0) at 37 °C for 24 h. For the latter, a compound (50 μL stock solution) was incubated with 50 μL buffer B at 37 °C for 24 h. Then samples were mixed with laemmli in ratio 1:1 and electrophoresed on 4%-20% NuBlu gel from NuSep using 10% tris-Glycine buffer containing 0.1% SDS. The results were imaged using 780 nm laser diode illumination and a CCD camera outfitted with appropriate filters to collect light emission at 830 nm.

### 3.7. Measurement of Fluorescence Intensity of HA-NIR-dyes (Quenching Effect)

Each HA-NIRdye conjugate was dissolved in buffer A to generate stock solution (20 μM). Fifty μL of each stock solution and 50 μL of buffer B were added to each well of a 96-well plate. The fluorescence intensity for each conjugate was measured by Odyssey Near-Infrared Imaging System (Lincoln, NE, USA) at channel 800 nm. The assay for each sample was repeated three times. The data was regressed using the software GraphPad Prism 4 (San Diego, CA, USA).

### 3.8. Measurement of Fluorescence Intensity of HA-NIR-Dyes by Degradation (Recovering Effect)

Each HA-NIRdye conjugate was dissolved in buffer A to generate stock solution (20 μM). Fifty μL of each stock solution and 50 μL of 5 units of hyaluronidase in buffer B were added in each well of a 96-well plate. The samples were incubated at 37 °C for 24 h. The fluorescence intensity for each conjugate was measured by Odyssey Near-Infrared Imaging System at channel 800 nm. The assay for each sample was repeated three times. The data was regressed using the software GraphPad Prism 4.

### 3.9. Michaelis-Menten Constant (Km) Determination of Bioactivable HA-NIRdyes

The HA-NIRdye conjugates (12 and 17% dye molar loading) were prepared at six different concentrations ranging from 20 to 120 μM in buffer A respectively. Hyal (30 units) was dissolved in 1 mL of buffer B. Each conjugate solution (50 μL) was mixed with Hyal (50 μL) in a well of a 96-well plate. The fluorescence intensities of each concentration were monitored by GENios Pro (San Jose, CA, USA) at 37 °C at 5 min interval for 30 min. The assay for each sample was repeated three times. The initial velocity (*v*) was determined by the slope of the fluorescence intensity versus time curve, whereby the fluorescence intensity for calculation was the average value of fluorescence intensities from the three assays. The Michaelis-Menten constant (K_m_) was determined by nonlinear regression with Michaelis-Menten equation using the software GraphPad Prism 4.

### 3.10. Kinetic Inhibition Assay of Bioactivable HA-NIRdye Conjugate with HA

Four of HA stock solutions were prepared in buffer A at different concentrations, 33, 66, 132 and 200 μM. Twenty assay solutions were prepared with HA-NIR-dye conjugate (12% dye molar loading) at five different concentrations ranging from 30 to 120 μM dissolved in each of the HA stock solution. Hyal (30 units) was dissolved in 1 mL of buffer B. Each assay solution (50 μL) was mixed with Hyal (50 μL) in a well of a 96-well plate. The fluorescence intensity of each solution was monitored by GENios Pro at 37 °C at 5 min interval for 30min. The assay for each sample was repeated three times. The initial rate (*v*) was determined by the slope of the fluorescence intensity versus time curve, whereby the fluorescence intensity used for the calculation was the average value of fluorescence intensities from the three assays. The data was analyzed using the software GraphPad Prism 4 with Lineweaver-Burk equation for competitive inhibition.

### 3.11. *In Vivo* Imaging Study with 1% or 17% Loading HA-NIRdye

Male and female SKH mice (4- to 6-week-old, 18-22 g) (Harlan, Indianapolis, IN, USA) were maintained in a pathogen-free mouse colony and fed with sterilized pellet chow (Harlan Sprague Dawley, Inc., Indianapolis, IN, USA) and sterilized water. The facilities are facility accredited by the American Association for Laboratory Animal Care (Accredited Facility Number: 876), and all experiments were performed in compliance with the guidelines of the Institutional Animal Care and Use Committee.

Five of SKH mice were used for each assay. For imaging study, mice were anesthetized by inhalation of isoflurane. A catheter was placed in the tail vein for intravenously injection. HA-NIRdye in 0.2 mL of saline was injected through the tail vein catheter. All mice received agents at equivalent of 5 nmol of dye. Mice were waked up between each imaging time point.

The imaging agents were excited by a diffused 80 mW and 785 nm laser source. The fluorescent emission signal was collected by an electron-multiplying charge-coupled device (EMCCD) camera (model PhotoMAX:512B, Princeton Instruments, Trenton, NJ, USA) at 830 nm wavelength. Image acquisition was accomplished using V++ software from Digital Optics (Auckland, New Zealand). Data processing and analysis were accomplished using ImageJ software (ImageJ 1.35, National Institutes of Health, Bethesda, MD, USA).

## 4. Conclusions

In this work, two kinds of fluorescent hyaluronan (HA-NIRdye) analogs were developed. *In vitro* measurements confirm the biological activity of the imaging agents while *in vivo* fluorescence imaging results show differences between quenched and unquenched agents that are consistent with degradation and clearance pathways. The imaging data demonstrate that HA-NIRdyes uptake and degradation in the body can be imaged by non-invasive molecular imaging techniques. Our results suggest that the HA-based NIR imaging agents may be utilized: (i) to follow HA uptake by a method which could be used for image guided resection and biopsy; and (ii) to detect Hyal associated with diseases as diagnostic markers for clinic applications. For example, enzyme sensitive fluorescent HAs may be able to be used for detecting bladder and prostate cancers, due to the fact that bladder cancer tissue expresses elevated levels of hyaluronidase compared with normal bladder tissue, and the level of hyaluronidase are also elevated in the urine of patients with high grade bladder cancer [29]. It is also found that hyaluronidase levels in prostate cancer tissue is significantly elevated and correlate well with the tumor grade [30]. In addition, the fluorescent HAs, possessing the properties of biocompatibility and non-immunogeneity of HA, may find biomedical application in the field of tissue engineering.
